# Optimizing Chitin Extraction from *Acheta domesticus*: A Sustainable Approach Using Two Ultrafine Grinding Techniques

**DOI:** 10.3390/ijms26072938

**Published:** 2025-03-24

**Authors:** Binqiao Yuan, Tinghao Yu, Junkui Huang, Xinrui Ren, Dawei Huang, Jinhua Xiao

**Affiliations:** College of Life Sciences, Nankai University, Tianjin 300071, China; 1120220611@mail.nankai.edu.cn (B.Y.); 1120220610@mail.nankai.edu.cn (T.Y.); 2120231459@mail.nankai.edu.cn (J.H.); 1120240815@mail.nankai.edu.cn (X.R.); huangdw@nankai.edu.cn (D.H.)

**Keywords:** superfine-grinding, house cricket, chitin, extraction, characterization

## Abstract

This research emphasizes the application of ultrafine grinding technologies to optimize the extraction process of chitin from house crickets (*Acheta domesticus*), aiming to establish a more sustainable and efficient production method. This study evaluates the extraction efficiency of two advanced ultrafine grinding techniques: (1) fluidized bed jet milling (FBJM) and (2) graded impact milling (GIM), alongside a traditional hand sieving method. A comprehensive analysis of the chemical composition of the extracted chitin was performed, measuring critical parameters such as moisture content, residual ash, and protein levels, while also assessing its physicochemical properties. The results demonstrate that the implementation of ultrafine grinding methods significantly enhances both the yield and purity of chitin, while also reducing raw material consumption. This highlights the potential of house crickets as a sustainable source of biomaterials. The findings provide essential theoretical insights and practical guidance for the future development and application of chitin derived from insects.

## 1. Introduction

In an age characterized by the continuous exploration of novel food resources, the *Acheta domesticus* (also known as the house cricket) is emerging as a new source of nutrition, steadily attracting increasing attention. As an edible insect, house crickets exhibit remarkable nutritional properties. They also exhibit enhanced feed conversion efficiency, reproductive ability, and environmental sustainability, which consistently captivate public interest [1]. Chitin is a natural polysaccharide synthesized by insects during their growth and development, primarily consisting of beta-1,4-linked N-acetyl-D-glucosamine (GlcNAc, NAG) units, and is predominantly located in the insect epidermis. It can be isolated from other body proteins through chemical processing [2]. The chitin derived from insect exoskeletons may exhibit minor structural variations when compared to that found in shrimp and crab shells. For instance, previous research indicates that chitin from black soldier flies possesses a honeycomb-like structure and contains catechol, which may influence its biological activity and physicochemical properties. Specifically, chitin extracted from *Gryllus bimaculatus* demonstrates superior fat absorption, emulsification, and emulsion stability, whereas chitin from *Acheta domesticus* shows increased solubility in water [3]. These findings suggest that crickets are a promising source of chitin. While prior studies have predominantly concentrated on the extraction yield of chitin, examining the effects of developmental stages or extraction methods [3,4,5], there is a lack of research on the impact of various pre-processing techniques. Traditional chitin extraction methods often involve significant chemical reagent usage and high energy consumption, leading to environmental pollution and resource depletion. Consequently, it is crucial to investigate pre-processing methods that enhance extraction efficiency and minimize material consumption.

House crickets exhibit great potential as a source of chitin [6,7,8], with a yield of 4.3% of extracted chitin from domestic crickets. In addition, the ash content was lower than that of shrimp and crab chitin, indicating better quality [9]. However, this yield remains comparatively modest. Although there have been many studies on the composition of chitin content in cricket epidermis, there is still relatively little research on extracting chitin from crickets as a whole, and there is no complete characterization study on the extracted chitin. Our previous investigations have demonstrated that ultrafine grinding could exert an influence on the physicochemical attributes of the house cricket, particularly the extraction of nutrients. The method of grinding and the extent of grinding also have an impact on the extraction rate of chitin. However, we have failed to pay attention to the variances in the physicochemical properties and the structural and functional characteristics of the chitin samples extracted. Our hypothesis is that the application of ultrafine grinding technology as a pre-treatment for domestic crickets can enhance the yield of chitin and facilitate the production of chitin products with higher purity. The application of ultrafine grinding technology can more efficiently utilize cricket materials, reduce material waste, and provide some ideas for optimizing insect chitin extraction.

To enhance production efficiency, this study employed fluidized bed jet milling (FBJM) and graded impact milling (GIM) as the primary techniques for chitin extraction. These advanced methods not only significantly increase extraction efficiency but also contribute to more sustainable production practices by minimizing raw material consumption. Ultrafine grinding technology is an advanced processing technique capable of reducing material particles to micro-, submicron-, over even nanometers- (100 μm~1 nm in size) [10]. In recent years, it has been widely applied and deeply studied in various fields such as medicine, food production, mining, and the chemical industry. This technology mainly uses mechanical forces such as impact, grinding, and shearing to progressively reduce the size of material particles. Common types of ultrafine grinding equipment include airflow grinders, impact mills, and vibrating mills. Airflow grinding utilizes high-velocity air streams to facilitate collisions and friction among material particles for effective grinding; impact mills rely on the motion of grinding bodies to exert force on the materials for crushing and pulverization [11]. Furthermore, this technology offers several benefits. It enhances the specific surface area and accelerates the dissolution speed of active ingredients, which improves their bioavailability. Additionally, it optimizes the physicochemical properties of functional materials, leading to a smoother food texture and greater stability of nutrient components. Lastly, it maximizes material utilization, thereby reducing resource waste [12].

This study investigated the extraction of chitin from ultrafine and regular powders of varying particle sizes using three distinct pre-treatment methods: fluidized bed jet milling, graded impact milling, and traditional manual sieving. The yields and purities of chitin obtained from these methods were compared. Furthermore, the physical and chemical properties of the extracted chitin were thoroughly characterized through techniques including scanning electron microscopy (SEM), Fourier-transform infrared spectroscopy (FTIR), X-ray diffraction (XRD), and 13C nuclear magnetic resonance (NMR). Additionally, the thermal stability of the chitin samples was assessed using thermogravimetric analysis (TGA). This study lies in the innovative application of two advanced ultrafine grinding techniques, fluidized bed jet milling (FBJM) and graded impact milling (GIM), to optimize chitin extraction from house crickets (*Acheta domesticus*), offering a more sustainable and efficient approach compared to traditional methods, and contributing to the development of insect-based biomaterials. The findings of this study provide a theoretical framework for optimizing chitin extraction processes from domestic crickets and offer insights into the potential applications of insect-derived bioactive compounds.

## 2. Results and Discussion

### 2.1. Index of Chitin Content in Crickets

The composition analysis of seven chitin samples is summarized in Table 1. Following the defatting process, the chitin content measured for ADFM-1, ADFM-2, ADGM-1, ADGM-2, ADM-1, ADM-2, and ADM-3 was found to be 4.62 ± 0.02, 10.17 ± 0.09, 6.46 ± 0.06, 4.46 ± 0.10, 9.61 ± 0.12, 9.34 ± 0.17, and 5.46 ± 0.63, respectively. Notably, ADFM-2 exhibited the highest chitin yield among all tested conditions, significantly surpassing the yields from other pre-treatment methods, thus confirming its effectiveness. In contrast, the GIM treatment caused structural degradation of the cricket powder, leading to a reduced chitin yield compared to that obtained through ADFM-2. The protein content of the chitin samples varied, with ordinary powder extracts showing values between 2.31 ± 0.50 and 3.08 ± 0.53, while ADFM and ADGM treatments resulted in lower protein residues ranging from 1.07 ± 0.46 to 1.90 ± 0.82 and 1.33 ± 0.46 to 2.14 ± 0.46, respectively. The ADFM-2 pre-treatment group demonstrated significantly lower residual protein and moisture content compared to the other groups. Additionally, the ash content varied across samples, with crude chitin typically containing impurities such as proteins and minerals, including calcium, phosphorus, and iron, which are challenging to eliminate [13]. Although both FBJM and GIM treatments effectively reduced protein residues, ADFM-2 also significantly decreased ash content. Samples processed with ADFM-2 and ADM-1 exhibited lower calcium and phosphorus levels, which may account for the viscosity differences observed among the treatments. Previous research has indicated that the concentration of residual minerals, particularly calcium and phosphate, plays a critical role in influencing these results [14].

Table 2 illustrates that the degree of acetylation (DA) for ADFM-1, ADFM-2, ADGM-1, ADGM-2, ADM-1, ADM-2, and ADM-3 was 64%, 52%, 48%, 48%, 49%, 69%, and 60% respectively. The viscosity values recorded were 417 ± 5.69 kDa for ADFM-1, 277 ± 4.04 kDa for ADFM-2, and lower values observed across the other samples. In this study, the values for extracted chitin samples were 100% higher than the theoretical values. Adam et al. reported the purity of chitin with DA values greater than 100%, possibly owing to the presence of some mineral residues in the chitin structure [15]. Using FTIR, we determined the degree of acetylation (DA) of commercial chitin, which was found to be 111. In comparison, the chitin extracted from house crickets exhibited a DA value closely resembling that of the commercial sample (DA: 95%). The comparable DA values between the chitin derived from domestic crickets and the commercial chitin indicate that the observed difference is unlikely to be attributed to the presence of residual proteins or other contaminants. Chitin processed using FBJM exhibited higher viscosity than those treated using GIM. However, the binding ability of water and oil in chitin was reduced post-FBJM and GIM processing.

The chemical extraction of chitin for industrial applications has been the subject of extensive research, particularly focusing on the pre-treatment of raw materials. In our study, which utilized ultrafine grinding technology to extract chitin from house crickets, samples processed under ADFM-2 treatment yielded the highest chitin while also exhibiting lower levels of ash, protein, and moisture compared to other treatments. This reduction in ash content suggests that the chitin extracted from house cricket materials is of a high grade and possesses enhanced solubility, making the residual ash content a crucial indicator of the demineralization process’s efficiency. Clearly, the ADFM-2 treatment performs better in this process.

As shown in Table 3, chitin samples recovered using ADM-1 treatment displayed the highest water binding capacity (WBC), which may be attributed to a greater quantity of chitin precursor fibers, enhancing the fiber’s surface area and its ability to form hydrogen bonds with water molecules [16]. Additionally, the fat binding capacity (FBC) was also highest in ADM-1 treated chitin, indicative of its potential utility in the food industry where fat absorption is critical. This enhanced functionality is likely due to the minimal mineral content in ADM-1 processed chitin, which facilitates better fat or oil capture [17]. Lower protein and mineral concentrations post-processing result in higher purity and quality of the chitin [18]. Therefore, to extract high-quality chitin from house crickets, it is essential to efficiently remove associated proteins and minerals.

### 2.2. Characterization of House Cricket Chitin

#### 2.2.1. FTIR

**Figure 1:**
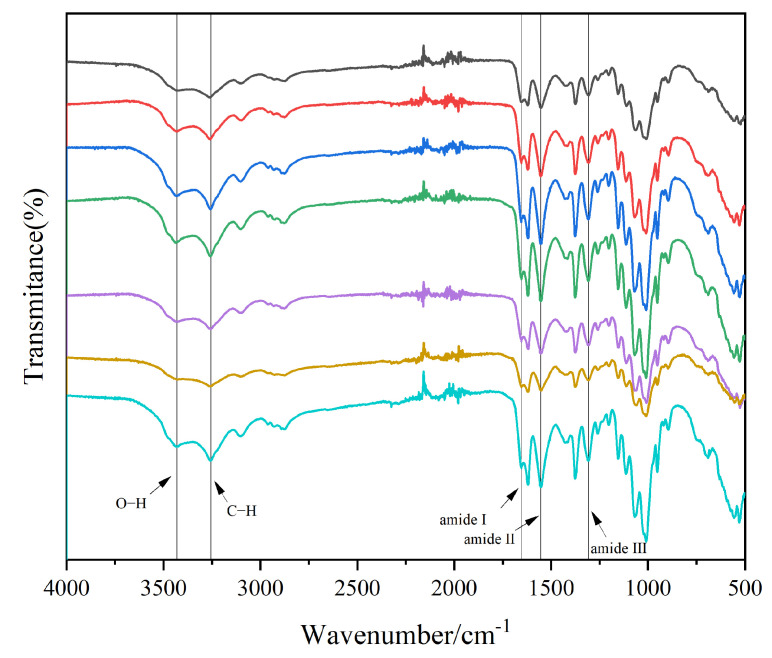
Infrared spectra of house cricket chitin samples (different pre-processing methods). The lines from top to bottom represent the following: black for ADFM1, red for ADFM2, blue for ADGM1, green for ADGM2, purple for ADM1, yellow for ADM2, and cyan for ADM3.

The FTIR spectra of house cricket chitin using different pre-treatment methods, as shown in Figure 1, closely resemble those of standard chitin, confirming the similarity between the cricket-derived chitin and the standard form. Characteristic absorption bands at 3436 cm^−1^ and 3259 cm^−1^ are attributed to O-H and N-H stretching vibrations, respectively. Furthermore, the peak at 3105 cm^−1^ is consistent with the presence of amide groups typically associated with α-chitin [19]. Three amide-related peaks are prominently observed: Amide I at 1655 cm^−1^, corresponding to C=O stretching, Amide II at 1545 cm^−1^, associated with N-H bending, and Amide III at 1315 cm^−1^, linked to C-N stretching. It is worth noting that amide I has two different bands (at approximately 1662 cm^−1^ and 1621 cm^−1^). In contrast, β-chitin has only one single peak (usually around 1621 cm^−1^) [20,21].

#### 2.2.2. X-Ray Diffraction

**Figure 2:**
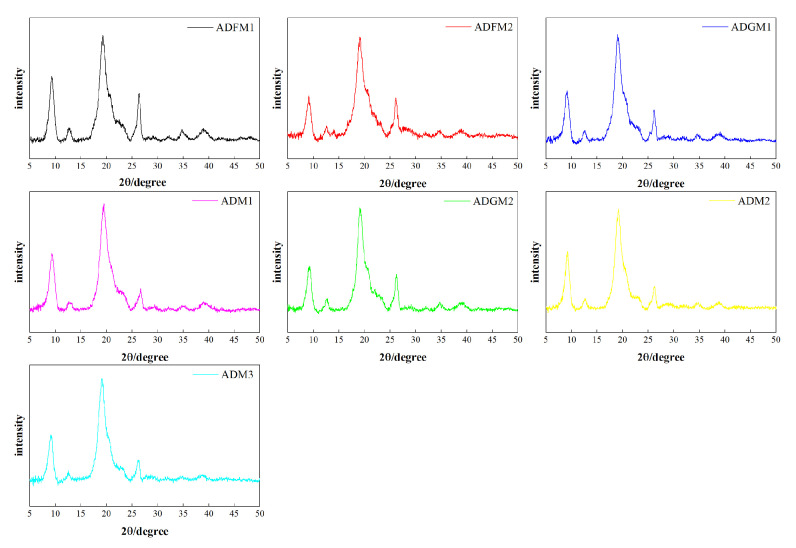
XRD patterns of house cricket chitin samples (different pre-processing methods).

As demonstrated in Figure 2, the X-ray diffraction (XRD) spectra of chitin processed under seven different conditions exhibit two prominent peaks at 9.2° and 19.1°, with four additional weaker peaks observed at approximately 12.5°, 23.3°, 26.1°, and 38.2°. These spectral features align closely with the XRD patterns of standard chitin. The samples processed by the FBJM method exhibited average crystallinity indices of 23 and 16, respectively. In contrast, those treated by the GIM method showed crystallinity indices of 10 and 24. Samples obtained through hand screening demonstrated crystallinity indices of 24, 20, and 20. The X-ray diffraction spectrum of chitin revealed two additional shoulder peaks at 12.5° and 26°, respectively, which were attributed to the binding of hydrogen molybdenum (iron) and sodium hydroxide with hydrogen molybdenum [22].

#### 2.2.3. 13C NMR

**Figure 3:**
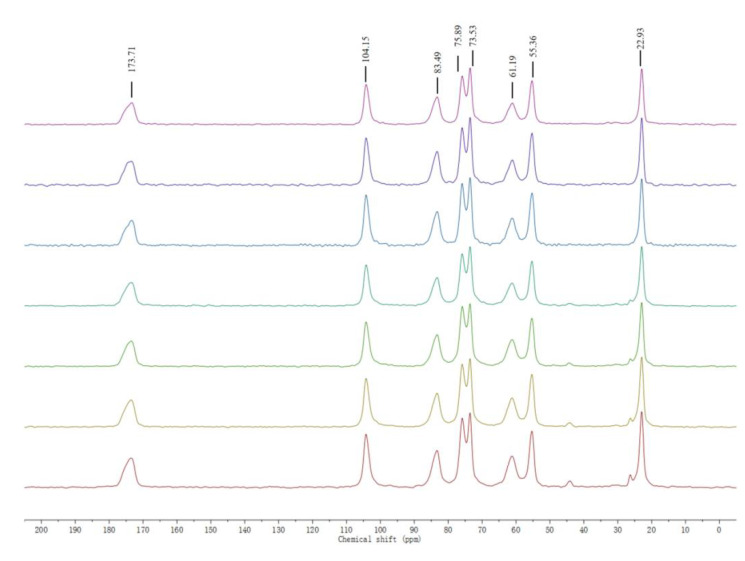
13C NMR spectra of house cricket chitin samples (different pre-processing methods). The lines, arranged from top to bottom, correspond to the following: the pink and purple lines are ADFM1, ADFM2; the blue and cyan lines are ADFM1, ADFM2; The green, yellow, and red lines represent ADM1, ADM2, and ADM3.

The structural characterization of chitin extracted from house crickets was performed using solid-state nuclear magnetic resonance (NMR) spectroscopy. In particular, 13C NMR spectroscopy offered detailed information on the chemical linkages within the chitin structure, while also enabling the quantification of acetyl content and the determination of the average acetylation degree [23]. As shown in Figure 3, the NMR spectra of chitin extracted from house crickets display eight distinct resonances, consistent with those observed in standard chitin. Six of these peaks correspond to the glucosamine ring between 45 and 115 ppm, while two peaks are attributed to the acetyl methyl group and carbonyl group, appearing around 25 ppm and 174 ppm, respectively. Notably, the spectrum of house cricket chitin reveals a splitting of the peak at 73 and 75 ppm, which is characteristic of α-chitin, whereas β-chitin typically shows a single peak near 75 ppm [24].

#### 2.2.4. SEM

**Figure 4:**
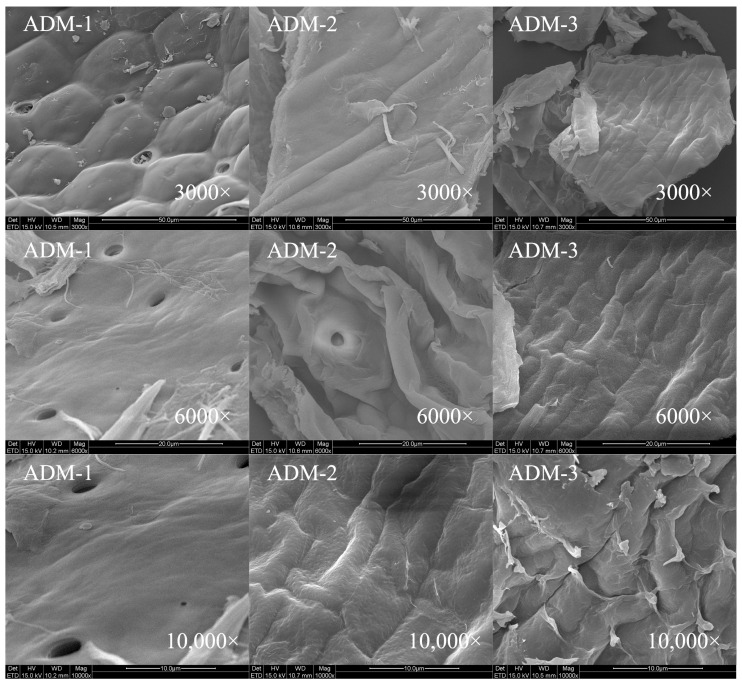
SEM images of house crickets chitin samples (different pre-processing methods). The three columns from left to right are ADM1, ADM2, and ADM3.

**Figure 5:**
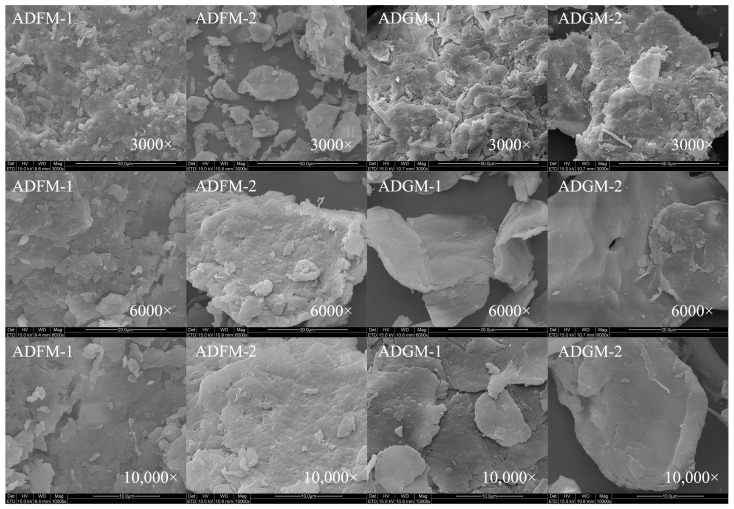
SEM of house crickets chitin samples (different pre-processing methods).

The surface morphology of chitin extracted from house crickets was analyzed to understand its structural variations. A honeycomb structure, depicted in Figure 4, was identified in the chitin samples obtained through hand sieving. As demonstrated in Figure 5, chitin processed via FBJM and GIM exhibited a dense, plate-like morphology. This structural feature closely parallels the characteristic architecture of chitin extracted from shrimp and crab shells, suggesting analogous hierarchical organization at the microscale. These morphological characteristics align with findings from Manel et al. [14], who observed similar traits in yellow mealworm chitin. In the scanning electron microscope images, it can be seen that the removal of calcium and phosphate during the extraction process leads to a gradual increase in the number of pores and an increase in volume. These images highlighted areas where the fiber surface appeared more porous and disordered, with visible aggregates of fibers following the removal of calcium and phosphorus [13]. Notably, chitin from shrimp features a surface interspersed with nanofibers, underscoring the importance of surface morphology in determining chitin’s applicability across various industries [19,20]. Additionally, fibrous and granular structures are prominent on the surface of crab shells [25], further emphasizing the role of morphology in functional performance.

In addition, it has been noted that chitin from different sources has different surface morphologies [26,27]. Previous studies have shown that chitin with surface pores exhibits improved metal ion absorption capacity. Additionally, this porous structure of house cricket chitin has great potential in the development of medical materials due to its excellent permeability.

#### 2.2.5. TGA

**Figure 6:**
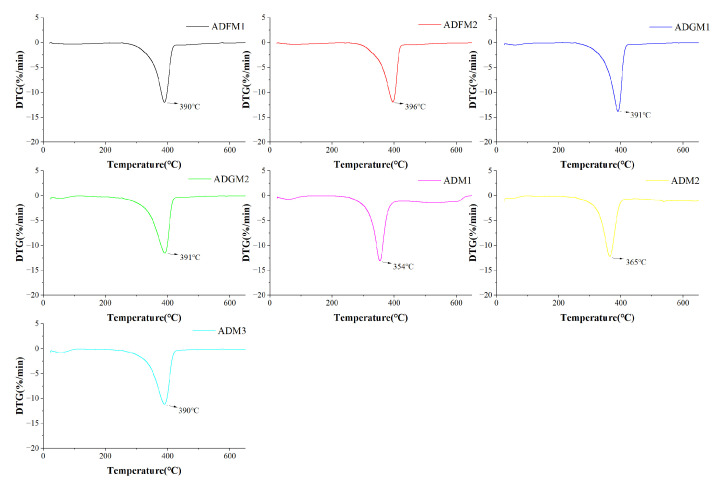
DTG curves of house crickets chitin samples (different pre-processing methods).

Thermogravimetric analysis revealed that chitin extracted from house crickets undergoes two primary degradation stages. Initially, the mass loss from 0 to 120 °C is attributed to the evaporation of water adsorbed within the chitin, as shown in Figure 6. Subsequently, a significant quality loss between 150 and 450 °C results from the breakdown of the sugar structures [28], dehydration of the sugar rings, and degradation of both acetylated and deacetylated chitin molecules, consistent with findings by Adam et al. [15].

The weight loss in the first degradation stage for chitin samples from seven cricket species varied slightly, showing a rate of 1.5% to 2.0% (Figure 6). The second stage exhibited a more substantial weight loss rate of 74% to 98%. Notably, the ADM-1 treatment displayed the highest rate at 98%, while ADFM-1 treatment showed the lowest at 74%. Moreover, TGA analysis indicates that chitin degradation initiates with water molecule evaporation between 50 and 100 °C, followed by molecular breakdown of the sugar structure from 250 to 500 °C. The highest thermal degradation temperature, observed after BJFM and GIM treatments, was 396 °C for ADFM-2 (Figure 6), demonstrating enhanced thermal stability of chitin pre-treatment.

## 3. Materials and Methods

### 3.1. Biological Materials

House crickets were acquired from Xuyang Insect Source Agricultural Development Co., Ltd., Zhengzhou City, Henan Province, China. They were fed a cereal feed powder consisting of wheat bran, soybeans, and corn, and were collected after seven weeks. The crickets were rinsed thoroughly with water, followed by drying in a microwave dryer set to 30 kW at a temperature of 60 °C for a duration of 20 min. After drying, they were stored at −20 °C in a freezer for later use.

### 3.2. Ultrafine Grinding

Microwave-dried house crickets were ground for 120 s using a high-speed grinder (YS-04B, Beijing, China). The resulting coarse powder was defatted using petroleum ether in a Soxhlet extractor until the solvent became clear. After defatting, the powder was dried in an air-drying oven (DHG-9240A, Shanghai, China) at 60 °C for 24 h. To achieve different particle sizes, the dried powder was sieved using standard meshes (0.38 mm and 0.15 mm) to prepare three distinct fractions: 40 mesh (96.9 μm), 100 mesh (84.8 μm), and 250 mesh (28.6 μm), which were designated as ADM-1 (96.9 μm), ADM-2 (84.8 μm), and ADM-3 (28.6 μm), respectively. Ultrafine cricket powders were produced using a graded impact mill (GIM) (ZJ-C50, Sichuan Zhongjin Powder Equipment Co., Ltd., Chengdu City, Sichuan Province, China) and a fluidized bed jet mill (FBJM) (ZJ-QLM50, Sichuan Zhongtou Powder Equipment Co., Ltd., Chengdu City, Sichuan Province, China), with processing parameters optimized to achieve different particle sizes. The powders obtained from the GIM were labeled ADGM-1 (8.4 μm) and ADGM-2 (20 μm), while those from the FBJM were designated as ADFM-1 (4 μm) and ADFM-2 (6.9 μm). The granularity of the ultrafine powders was primarily determined by adjusting the frequency parameters of the classifier and the induced draft fan of the mills. Specifically, the classifier frequencies for ADGM-1, ADGM-2, ADFM-1, and ADFM-2 were set at 20 Hz, 40 Hz, 15 Hz, and 50 Hz, respectively, while the frequencies for the induced draft fans were 30 Hz, 38 Hz, 50 Hz, and 20 Hz, respectively. After processing, the powders were stored in a −20 °C refrigerator.

### 3.3. Chitin Extraction

House cricket powder was treated with 1 M hydrochloric acid and continuously stirred for 30 min at 60 °C using a magnetic stirrer to facilitate the elimination of mineral impurities from the house cricket powder. Following this, the sample was thoroughly rinsed with distilled water until a neutral pH was achieved. Then, in order to remove the protein from house crickets, 3 M sodium hydroxide solution was used and treated on a magnetic stirrer for 2 h (80 °C), and the deproteinized samples were washed. The final decolorization step involves treating the sample with a 10% (*v*/*v*) hydrogen peroxide solution at 50 °C for 1 h [29]. The treated sample was dried in an oven at 60 °C for 48 h to yield chitin. The chitin yield was calculated using the following formula:

Chitin yield (%) = weight of chitin/weights of the insect powder × 100

### 3.4. Chemical Analysis

According to the AOAC method, the chemical composition of chitin extracted from house cricket powder with different particle sizes was determined [30]. The moisture content is determined by drying the sample in a 105 °C oven, while the ash content is measured by heating the sample in a 550 °C muffle furnace. The residual protein content in chitin was determined using the Kjeldahl nitrogen determination method. The specific operation is to treat 0.5 g of the sample with 50 mL of 10% (*w*/*v*) sodium hydroxide solution at 90 °C for 2 h, centrifuge, and then determine the residual protein in the supernatant using the Kjeldahl method.

### 3.5. Viscosity Measurement

Dissolve 0.03 g of house cricket chitin sample in a 5% (*w*/*v*) LiCl/DMAc solution at 1 dL, and measure the viscosity of the resulting chitin solution using an Ubbelohde capillary viscometer (ThermoCap II, Fungilab, Beijing, China) at 25 °C. This measurement was repeated three times to ensure reliability. The viscosity values (MVs) were then calculated using the Mark–Houwink–Sakurada equation [31]:

[η]=KMv

In this context, [η] represents the intrinsic viscosity of the chitin sample, with K being 7.6 × 10^−4^ dL/g and α equal to 0.76.

### 3.6. Water Oil Binding Ability

To evaluate the binding ability of house cricket chitin samples to water or soybean oil, 10 mL of water or oil was added to a centrifuge tube containing 0.5× *g* of the sample. The mixture was then vortexed for 1 min and allowed to stand at room temperature for 30 min, with intermittent agitation five times at 10 min intervals. Afterward, the mixture was centrifuged at 3200 rpm for 25 min. The supernatant was carefully removed, and the tube was reweighed. The amount of water or oil retained by the sample was calculated using the following formula [32]:

$$\mathrm{WBC}\left(\%\right)=\frac{\mathrm{Water}\mathrm{bound}\mathrm{weight}\mathrm{sample}}{\mathrm{weight}\mathrm{of}\mathrm{chitin}}\times 100$$

$$\mathrm{FBC}\left(\%\right)=\frac{\mathrm{Fat}\mathrm{bound}\mathrm{weight}\mathrm{sample}}{\mathrm{weight}\mathrm{of}\mathrm{chitin}}\times 100$$

### 3.7. Fourier-Transform Infrared Spectroscopy (FTIR)

The functional groups in the cricket chitin samples were analyzed using Fourier-transform infrared (FTIR) spectroscopy. The samples were mixed with anhydrous potassium bromide and compressed into pellets under a vacuum for measurement. The Nicolet iS50 spectrometer (Thermo Fisher Scientific, Madison, WI, USA) was used to record FTIR spectra in the wavenumber range of 500 to 4000 cm^−1^ with a resolution of 2 cm^−1^. The resulting spectral data were processed using OMNIC analysis software 8.2 (Thermo Fisher Scientific, Madison, WI, USA). The degree of acetylation (DA) of chitin was calculated using the following equation [33]:

DA%=A1320A1420

### 3.8. Nuclear Magnetic Resonance Spectroscopy(13C)

Spectral analysis of cricket chitin was performed using a Bruker WB 400 spectrometer with cross polarization (CP) technique. This method helps to transfer magnetization from proton rich nuclei (1H) to nuclei with lower abundance, such as 13C and 15N, thereby shortening the testing time and improving testing efficiency by collecting stronger nuclear signal intensity.

### 3.9. X-Ray Diffraction (XRD)

The structural characteristics and crystallinity of the extracted cricket chitin were evaluated using an X-ray diffractometer (Brooke X-ray D8, Karlsruhe, Germany). X-ray diffraction (XRD) data were collected using nickel-filtered Cu-Kα radiation. The diffraction patterns were recorded over a 2θ range of 5° to 50°, with a step size of 0.02° and a counting time of 0.1 s per step. Crystallinity analysis was performed by processing the XRD patterns with HighScore Plus software 3.0.5 (Philips Analytical, Eindhoven, The Netherlands).

### 3.10. Scanning Electron Microscopy (SEM)

The surface morphology of extracted cricket chitin was examined using a scanning electron microscope (SEM) (QUANTA 200, FEI, Hillsboro, OR, USA) [34]. Microscopic images captured the detailed features. Prior to observation, the samples were prepared by undergoing a gold sputtering process to enhance electron conductivity.

### 3.11. Thermogravimetric Analysis (TGA)

The thermal degradation behavior of chitin samples with varying particle sizes was analyzed using thermogravimetric analysis (TGA) performed on NETZSCH equipment (TG 209 F3 Tarsus—METTLER TOLEDO, Selb, Germany) [35]. Take about 20 milligrams of sample for testing, and heat the test range from 25 °C to 650 °C (at a speed of 10 °C/min). Each chitin sample was measured in triplicate to ensure reproducibility.

### 3.12. Statistical Analysis

All measurements were conducted in triplicate and biologically replicated to ensure the reliability of the results. Statistical analyses were performed using IBM SPSS Statistics (Version 25, IBM, Armonk, NY, USA) and Origin 2021 software (OriginLab Corporation, Northampton, MA, USA) to provide a comprehensive evaluation of the data.

## 4. Conclusions

The chitin extracted from domestic crickets was characterized as alpha chitin. Our findings validated the hypothesis that employing ultrafine grinding technology significantly enhances both the yield and purity of chitin derived from domestic crickets. The ADFM-2 method yielded the highest chitin content, while simultaneously reducing the concentrations of protein, moisture, and minerals, and improving thermal stability. Consequently, ADFM-2 has been established as the most efficient technique for obtaining high-quality chitin. Although the viscosity of chitin obtained via ADFM-2 is lower than that of ADFM-1, this characteristic facilitates easier processing and dissolution, thereby enhancing biocompatibility and functional attributes. The experimental data suggest that the FBJM method enables the extraction of chitin with minimal material usage and high purity within a short timeframe, thus significantly optimizing resource utilization. The pre-treatment strategy developed by our research team not only enhances chitin yield and purity but also offers a novel approach for the sustainable exploitation of insect resources. By converting crickets into valuable biomaterials, this study contributes to resource recycling and supports sustainable development objectives. Furthermore, the distinctive physical and chemical properties of cricket-derived chitin underscore its potential as a promising biotechnological resource for bioenergy production from insects.

## Figures and Tables

**Table 1:** Content analysis of house cricket chitin samples (different pre-processing methods).

	Chitin Yield (%)	Moisture (%)	Protein (%)	Ash (%)
ADFM-1	4.62 ± 0.02 ^e^	5.90 ± 0.39 ^bc^	1.90 ± 0.82 ^ab^	1.17 ± 0.16 ^c^
ADFM-2	10.17 ± 0.09 ^a^	4.92 ± 0.28 ^a^	1.07 ± 0.46 ^a^	0.17 ± 0.09 ^ab^
ADGM-1	6.46 ± 0.06 ^c^	4.87 ± 0.28 ^a^	1.33 ± 0.46 ^ab^	0.55 ± 0.15 ^b^
ADGM-2	4.46 ± 0.10 ^e^	6.10 ± 0.39 ^c^	2.14 ± 0.46 ^bc^	1.39 ± 0.17 ^cd^
ADM-1	9.61 ± 0.12 ^b^	5.35 ± 0.29 ^b^	2.31 ± 0.50 ^bc^	0.14 ± 0.04 ^a^
ADM-2	9.34 ± 0.17 ^b^	8.30 ± 0.34 ^d^	2.97 ± 0.47 ^c^	0.37 ± 0.11 ^ab^
ADM-3	5.46 ± 0.63 ^d^	5.87 ± 0.16 ^bc^	3.08 ± 0.53 ^c^	1.57 ± 0.47 ^d^

Data are expressed as mean ± SD; Different lowercase letters within the same column indicate significant differences (*p* < 0.05) by Duncan’s multiple range test.

**Table 2:** Physical parameters of house cricket chitin samples (different pre-processing methods).

	DA (%)	Viscosity (kDa)	Crystallinity (%)
ADFM-1	115	417 ± 5.69 ^a^	23
ADFM-2	121	277 ± 4.04 ^b^	16
ADGM-1	124	262 ± 4.73 ^c^	10
ADGM-2	122	245 ± 4.93 ^d^	24
ADM-1	119	218 ± 3.06 ^e^	24
ADM-2	109	220 ± 7.10 ^e^	20
ADM-3	118	238 ± 2.52 ^d^	20

Data are expressed as mean ± SD; Different lowercase letters within the same column indicate significant differences (*p* < 0.05) by Duncan’s multiple range test.

**Table 3:** Binding ability of house cricket chitin samples (different pre-processing methods).

	WBC (%)	FBC (%)
ADFM-1	304.91 ± 1.67 ^g^	294.03 ± 2.11 ^e^
ADFM-2	557.68 ± 5.99 ^e^	649.16 ± 10.00 ^b^
ADGM-1	494.40 ± 7.56 ^f^	475.82 ± 17.39 ^d^
ADGM-2	603.10 ± 11.51 ^d^	534.57 ± 26.58 ^c^
ADM-1	1040.88 ± 31.10 ^a^	715.92 ± 3.30 ^a^
ADM-2	915.34 ± 12.41 ^b^	709.30 ± 12.57 ^a^
ADM-3	814.85 ± 14.32 ^c^	715.45 ± 7.00 ^a^

Data are expressed as mean ± SD; Different lowercase letters within the same column indicate significant differences (*p* < 0.05) by Duncan’s multiple range test.

## Data Availability

Data are contained within the article.
